# Effect of cultivation mode on bacterial and fungal communities of *Dendrobium catenatum*

**DOI:** 10.1186/s12866-022-02635-6

**Published:** 2022-09-21

**Authors:** Mingmin Zhu, Huihui Chen, Jinping Si, Lingshang Wu

**Affiliations:** State Key Laboratory of Subtropical Silviculture, Zhejiang A & F University, and Dendrobium catenatum Engineering and Technical Research Center of State Forestry Administration, Lin’an, 311300 People’s Republic of China

**Keywords:** *Dendrobium catenatum*, Cultivation mode, Bacterial community, Fungal community, Plant organ, Main chemical components

## Abstract

**Background:**

The orchid growth and development often associate with microbes. However, the interaction between plant performance and microbial communities within and surrounding plants is less understood. *Dendrobium catenatum*, which used to be an endangered orchid species, has become a billion dollar industry in China. Simulated natural cultivation modes, such as living tree epiphytic (LT) and cliff epiphytic (CE) cultivations, improve the production or quality of *D. catenatum* and contribute to the development of *D. catenatum* industry. In a previous study, morphological characteristics, anatomical structure, and main bioactive components (polysaccharides and ethanol-soluble extractives) of *D. catenatum* grown under LT and CE significantly differed from a facility cultivation mode, pot (PO) cultivation, were observed. Whether cultivation mode affects bacterial and fungal communities of *D. catenatum*, thereby affecting the chemical quality of this plant, need to be explored.

**Results:**

Both three plant organs (leaf, stem, and root) and cultivating substrates obtained under three cultivation modes: living tree epiphytic (LT), cliff epiphytic (CE), and pot (PO) cultivation were examined by adopting high-throughput sequencing methods. Subsequently, bacterial and fungal correlations with *D. catenatum* main chemical components, stem polysaccharides and ethanol-soluble extractives and leaf phenols and flavonoids, were elucidated. The results showed that microbial communities of the plants and substrates are both influenced by the cultivation mode. However, the plants and their cultivating substrates exhibited different patterns of bacterial and fungal composition, with clearly distinguished dominant bacterial groups, but shared dominance among fungal groups. Bacteria and fungi differed in abundance, diversity, and community structure, depending on the cultivation environment and plant organ. Both bacterial and fungal communities were affected by cultivation mode and plant organ. In both plants and substrates, PO bacterial and fungal community structure differed significantly from those of LT and CE modes. Bacterial and fungal community structure differed significantly between roots and the other two plant organs examined (stems and leaves). Several bacteria and fungi were positively correlated with main chemical components in *D. catenatum*.

**Conclusions:**

The findings indicate that microbial communities of the plants and substrates were both influenced by the cultivation mode and plant organ, and some of them were positively correlated with main chemical components in *D. catenatum*. The research would enhance our understanding of interactions between *Dendrobium* and the microbial environment, and to provide a theoretical basis for the development of improved *D. catenatum* cultivation methods.

**Supplementary Information:**

The online version contains supplementary material available at 10.1186/s12866-022-02635-6.

## Introduction

Plants host a diverse community of microorganisms including fungi and bacteria. Fungal and bacterial communities play multiple roles in plant growth and can provide protection from invading pathogens. Microbes within the rhizosphere can also affect plant nutrient acquisition, health, and productivity [1]. Plant microbiome studies have confirmed that plants harbor distinct microbiota that represent subsets of those found in the ambient environment [2]. Microbial communities in endosphere and rhizosphere have been shown to link to multiple host traits, e.g. growth, metabolism and resistance [3–5]. However, our understanding of the interaction between plant traits and microbial communities within and surrounding plants remains limited.

Orchidaceae is the largest and the most diverse family of angiosperms [6, 7], two-thirds of which are epiphytes that are mainly distributed in tropical forests [8]. Orchids are unique among plants in the manner of their assimilation of nutrients, which often necessitates relationships with fungi [9]. Mycorrhizal fungi, which are important for seed germination and plant survival [10], are also important for orchid health, maintenance, and growth. Non-mycorrhizal endophytic fungi in orchids, another major group of ubiquitous plant symbionts, play physiological roles in promoting seed germination and plant growth, and are potential sources of novel bioactive compounds [11, 12]. Endophytic bacteria have essential functions in the life history of orchids, influencing the formation of mycorrhiza and stability of plant–fungus interactions and promoting orchid growth and development [13–15]. Many previous studies have investigated microbial communities using a culture-dependent approach. However, most microbes cannot be cultured [16]. The recent development of culture-independent, high-throughput molecular approaches has transformed our understanding of plant-microbe relationships in both the host and the environment [2]. However, few studies have applied these methods to investigate both bacterial and fungal communities in orchids.

*Dendrobium* is a large genus of tropical epiphytic orchids, which are valued for their medicinal bioactive compounds [17]. *Dendrobium catenatum* Lindl. (*D. officinale*) has been described as “the first of the nine Chinese fairy herbs”, and is recorded in the Chinese Pharmacopoeia [18] for its unique effects on nourishing stomach, relieving throat inflammation and fatigue, promoting body fluid secretion, reducing peripheral vascular obstruction, preventing cataract development, and promoting the immune system [19]. Stem polysaccharides and ethanol-soluble extractives are its main functional chemical components [20]. Phenolics, e.g., flavonoids, bibenzyls, coumarins, are primary bioactive components in leaves [21]. *D. catenatum* has been listed as endangered in the Chinese Plant Red Book since 1987 due to its slow growth in the wild, limited distribution, and self-sterility [22]. Like many epiphytes, *D. catenatum* do not depend on soil for water and nutrient absorption and grow in the host trees without obvious use of the nutrient source of the living host [23]. Therefore, simulated natural cultivation modes, such as living tree epiphytic (LT) and cliff epiphytic (CE) cultivations, are primarily applied for large scale production of *D. catenatum* [24]. The method for LT is tying *D. catenatum* to a tree trunk and/or branches. The tree then provides the necessary shade and support for the herb to grow. The method for CE is planting *D. catenatum* on a rock wall at an angle of 85-90° to avoid exposure to sunlight in cliff cultivation process. These cultivation modes can improve the production or quality of *D. catenatum* by adopting highly efficient ecological cultivation involving natural growth conditions.

In a previous study, we found significantly different morphological characteristics, anatomical structure, and main bioactive components (polysaccharides and ethanol-soluble extractives) of *D. catenatum* ‘Jingpin Tianmushan’ (C13) grown under three different cultivation modes: LT, CE and pot (PO) cultivation, which may be due to different cultivation environments [25, 26]. Studies have shown that the content of metabolites from the same medicinal plant species can be different depending on their location of cultivation, which could in part be related to different composition in their associated microbes when grown at different sites [3, 27]. The variations in the main bioactive components of *D. catenatum* influenced by cultivation environment may also be partially impacted by changes in the microbial community either in the cultivation environment or in the plant endophytic compartment. Herein, we examined (1) whether and how bacterial and fungal communities within and surrounding *D. catenatum* ‘Jingpin Tianmushan’ would be influenced by cultivation mode; and (2) whether the fungal and bacterial communities from its cultivating substrates would influence plant-associated bacterial and fungal communities, which of *D. catenatum* ‘Jingpin Tianmushan’ thereby contributing to the variations of the plant main bioactive components (i.e., polysaccharides, ethanol-soluble extractives, phenols, and flavonoids). We applied high-throughput sequencing methods to sequence partial bacterial 16S rDNA and fungal ITS rDNA genes to compare the bacterial and fungal communities in *D. catenatum* ‘Jingpin Tianmushan’ and its cultivating substrates from three artificial cultivation modes (LT, PO, and CE), and to elucidate their correlations with the main chemical components of *D. catenatum*. The objective of this study was to enhance our understanding of the microbial environment influence on the performance of *Dendrobium*, and to provide a theoretical basis for the development of improved *D. catenatum* cultivation methods.

## Results

### Effects of cultivation mode on microbial diversity

In total, 1,696,003 and 1,240,820 quality-filtered and chimera-checked 16S/ITS rRNA gene sequences were obtained, with average lengths of 189 and 267 bp across all samples, respectively. In total, 5883 bacterial and 582 fungal OTUs were obtained from the 42 samples. Among these, 5478 bacterial and 494 fungal OTUs were obtained from the plant samples, and 3646 bacterial and 498 fungal OTUs were obtained from the substrate samples.

**Fig. 1:**
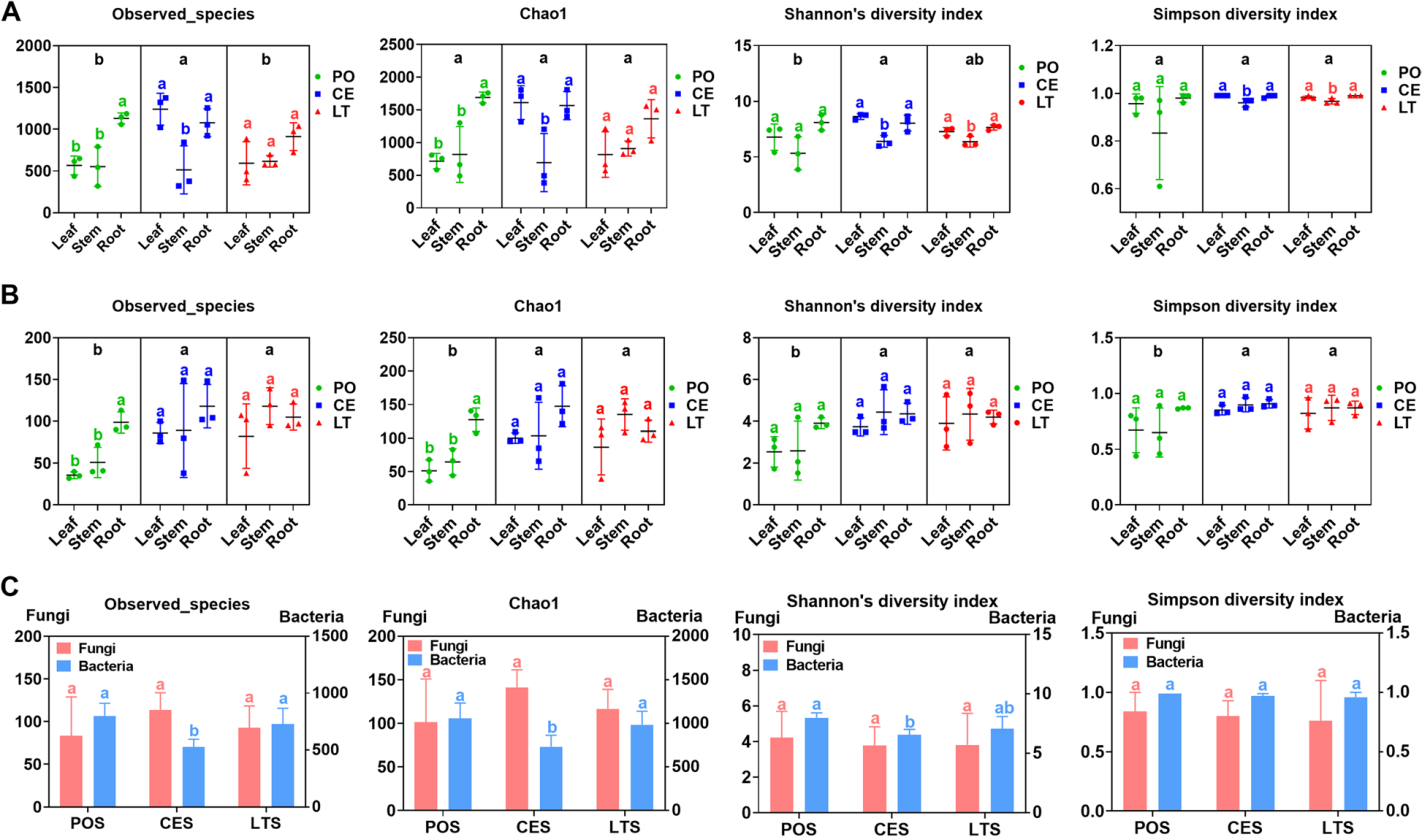
Estimated values of bacterial (**A**) and fungal (**B**) community relative abundances and diversity index for *D. catenatum* plants from three cultivation modes and their responding substrates (**C**). Significant differences are indicated by different letters

Bacterial and fungal community abundances (observed species and Chao1) and diversity (Shannon and Simpson) index values were compared for the plant samples. The abundance and diversity indexes for bacterial communities in plants both had no difference among cultivation modes (*P* > 0.05, Fig. 1A). Bacterial abundance indexes were significantly higher in roots than in leaves and stems in PO, and significantly higher in leaves and roots than in stems in CE (*P* < 0.05, Fig. 1A). In LT and CE, diversity index values were significantly higher in leaves and roots than in stems. Fungal community observed species and diversity were significantly higher in CE and LT plants than in PO plants (*P* < 0.05, Fig. 1B), and fungal abundances of roots were significantly higher than those of leaves and stems and fungal diversity of roots and stems were significantly higher than those of leaves in PO. Also, fungal abundances of roots were significantly higher than those of leaves in CE (*P* < 0.05, Fig. 1B).

Bacterial and fungal community abundances and diversity index values were also compared among cultivation substrates (Fig. 1C). Abundances and Shannon diversity index values for substrate bacterial communities differed significantly among the three cultivation modes, whereas those for substrate fungal communities did not (Fig. 1C). Bacterial community abundance and diversity index was significantly lower in CES than in POS (*P* < 0.05).

**Fig. 2:**
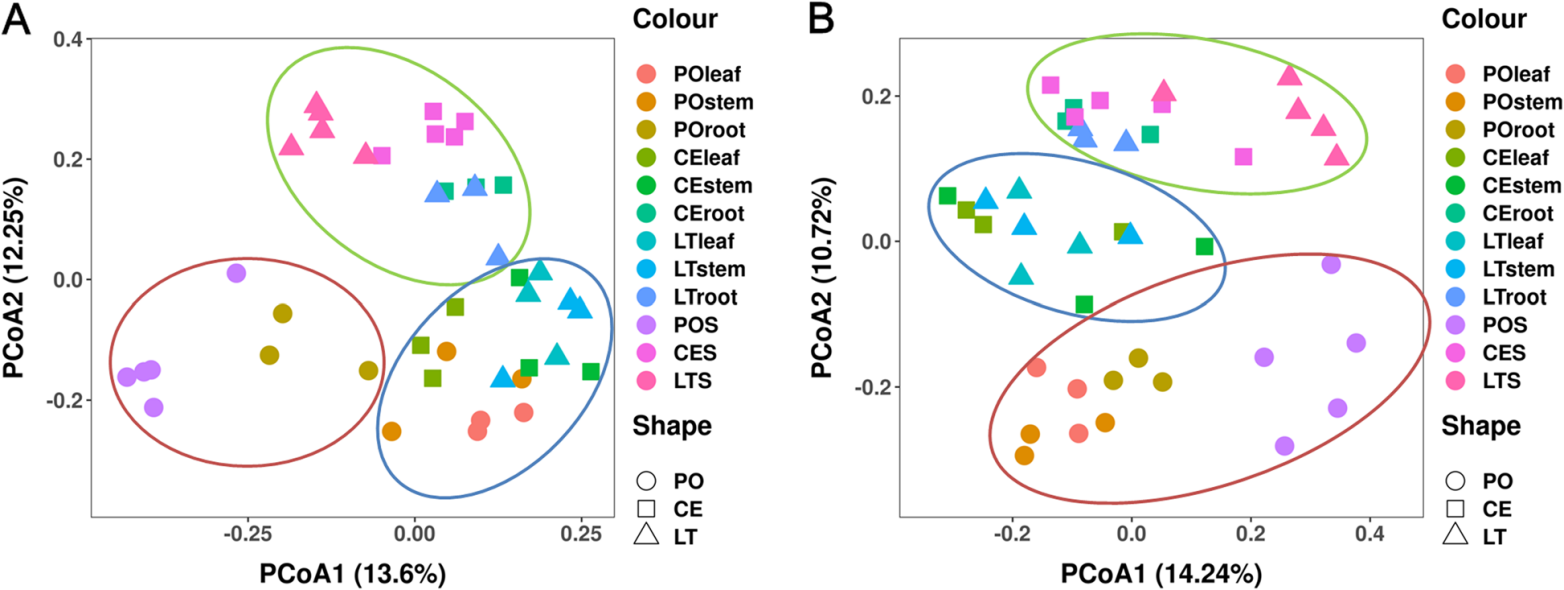
Principal coordinates analysis result of bacterial (**A**) and fungal (**B**) communities based on unweighted unifrac at phylum and class levels

PCoA analyses showed clear clustering of bacterial and fungal communities according to cultivation mode and plant organ. The bacterial community compositions of *D. catenatum* stems and leaves were clustered together in all three cultivation modes (Fig. 2A). The bacterial community compositions of roots showed similar clustering in CE and LT, and were close to their cultivation substrates. The bacterial community compositions of roots showed similar clustering for PO and POS. PCo1 and PCo2 explained 13.60 and 12.25%, respectively, of the total variation.

The fungal community compositions of leaves, stems, and roots from PO and POS were clearly distinguished from the other two modes and their cultivation substrates, and those of roots were close to their related cultivation substrates (Fig. 2B). The fungal compositions of stems and leaves were relatively close in CE and LT, whereas those of roots were close to their cultivation substrates. PCo1 and PCo2 explained 14.24 and 10.72%, respectively, of the total fungal community variation.

### Effects of cultivation mode on microbial community composition

**Fig. 3:**
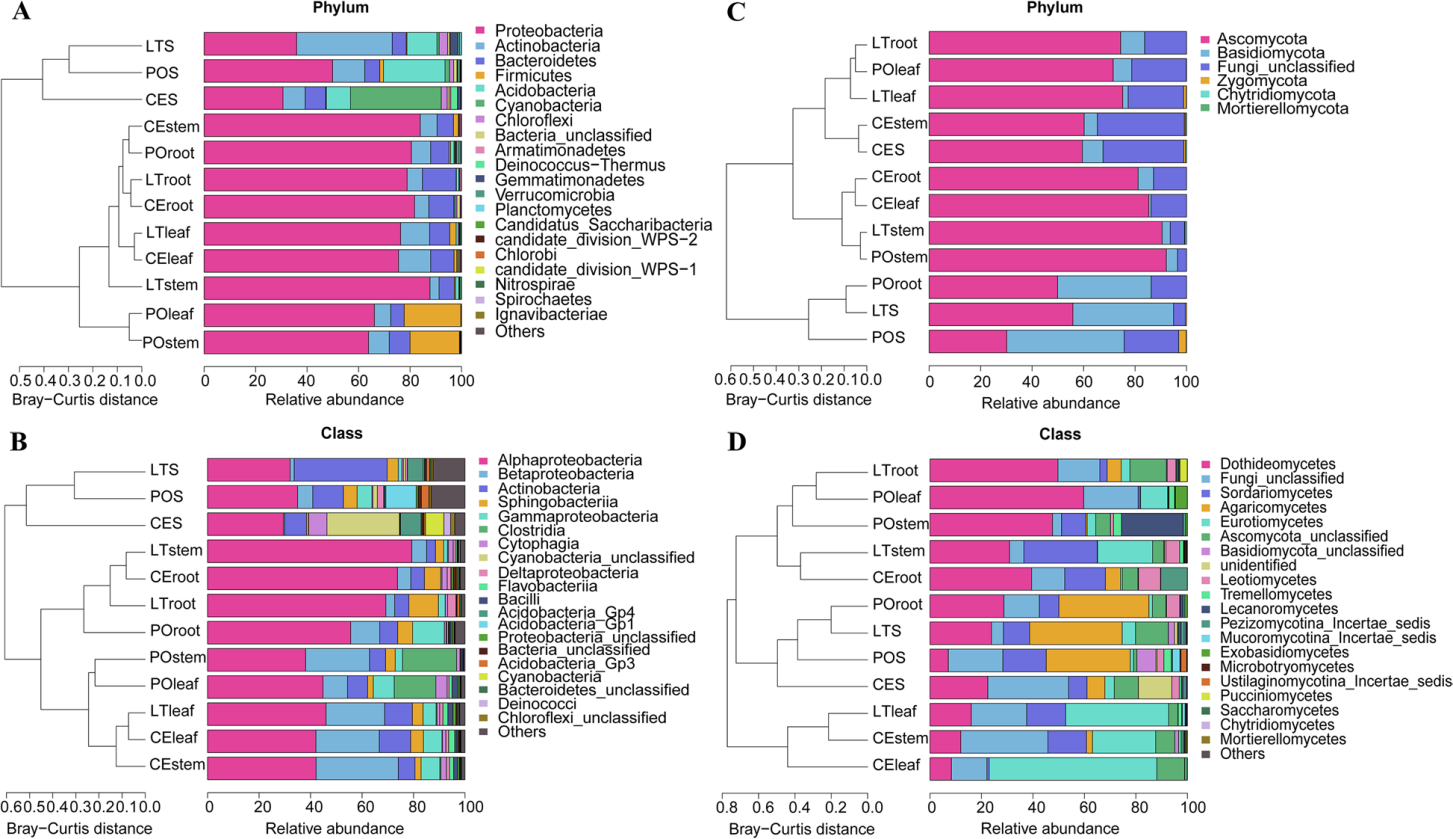
Relative abundances of bacterial (**A, B**) and fungal (**C, D**) groups for *D. catenatum* plants from three cultivation modes and their responding substrates

Bacterial communities differed significantly between the plants and their substrates (Fig. 3A, B) and among the three substrates. The predominant bacterial phyla in POS were Proteobacteria (49.92%), Acidobacteria (23.90%), Actinobacteria (12.49%), and Bacteroidetes (5.81%); those in LTS were Actinobacteria (37.71%), Proteobacteria (35.94%), Acidobacteria (11.62%), and Bacteroidetes (5.45%); and those in CES were Cyanobacteria (35.24%), Proteobacteria (30.61%), Acidobacteria (9.41%), Actinobacteria (8.66%), and Bacteroidetes (7.96%). The predominant groups of bacterial communities in plants were largely consistent among the three cultivation modes. The bacterial phyla with the highest relative abundance were Proteobacteria (66.89–87.76%), Actinobacteria (3.49–12.49%), Bacteroidetes (5.23–13.09%), and Firmicutes (0.09–22.00%). However, differences in relative abundances were detected among the three cultivation modes (Fig. 3A). For example, the relative abundance of Firmicutes was significantly higher in leaves (19.16%) and stems (22.00%) of PO than in those of the other modes.

The predominant fungal communities were largely consistent among the plants and substrates. The predominant fungal phyla were Ascomycota and Basidiomycota (Fig. 3C). However, differences in the relative abundances of fungal communities were observed at lower taxonomic levels (Fig. 3D). For example, the relative abundances of Agaricomycetes (Basidiomycota) were higher in the three substrates and plant roots. Among the three substrates, POS and LTS had higher relative abundances of Agaricomycetes (POS: 32.66%, LTS: 35.94%) than CES (6.95%). Among root samples from the three modes, those from PO had the highest relative abundance of Agaricomycetes (34.95%), whereas that from CE had the lowest (5.87%). Among the three cultivation modes, leaves and stems from CE had the highest relative abundances of Eurotiomycetes (leaf: 65.12%, stem: 24.55%). The relative abundance of Leotiomycetes was highest in roots in both CE and LT (CC: 5.17%, LT: 8.52%) and in stems (5.32%) and roots (3.33%) in PO.

**Fig. 4:**
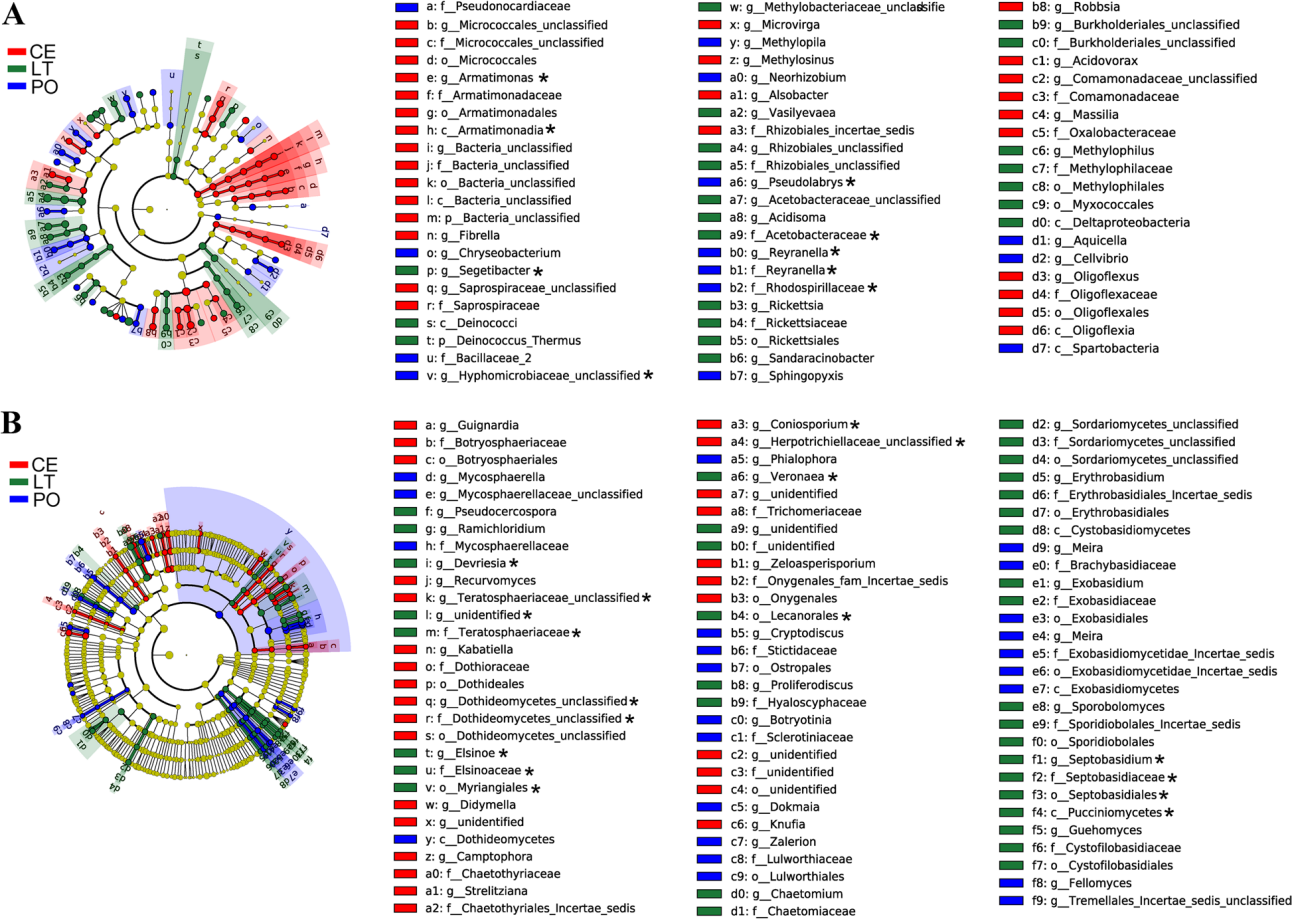
Cladograms showing the phylogenetic distribution of the bacterial (**A**) and fungal (**B**) lineages associated with *D. catenatum* from three different modes. Circles indicate phylogenetic levels from domain to genus. The diameter of each circle is proportional to the abundance of the group. Different-colored regions represent different cultivation modes. CE: cliff epiphytic cultivation, LT: living tree epiphytic cultivation, PO: Pot cultivation. *: the biomarkers shared by the plants and the substrates

**Fig. 5:**
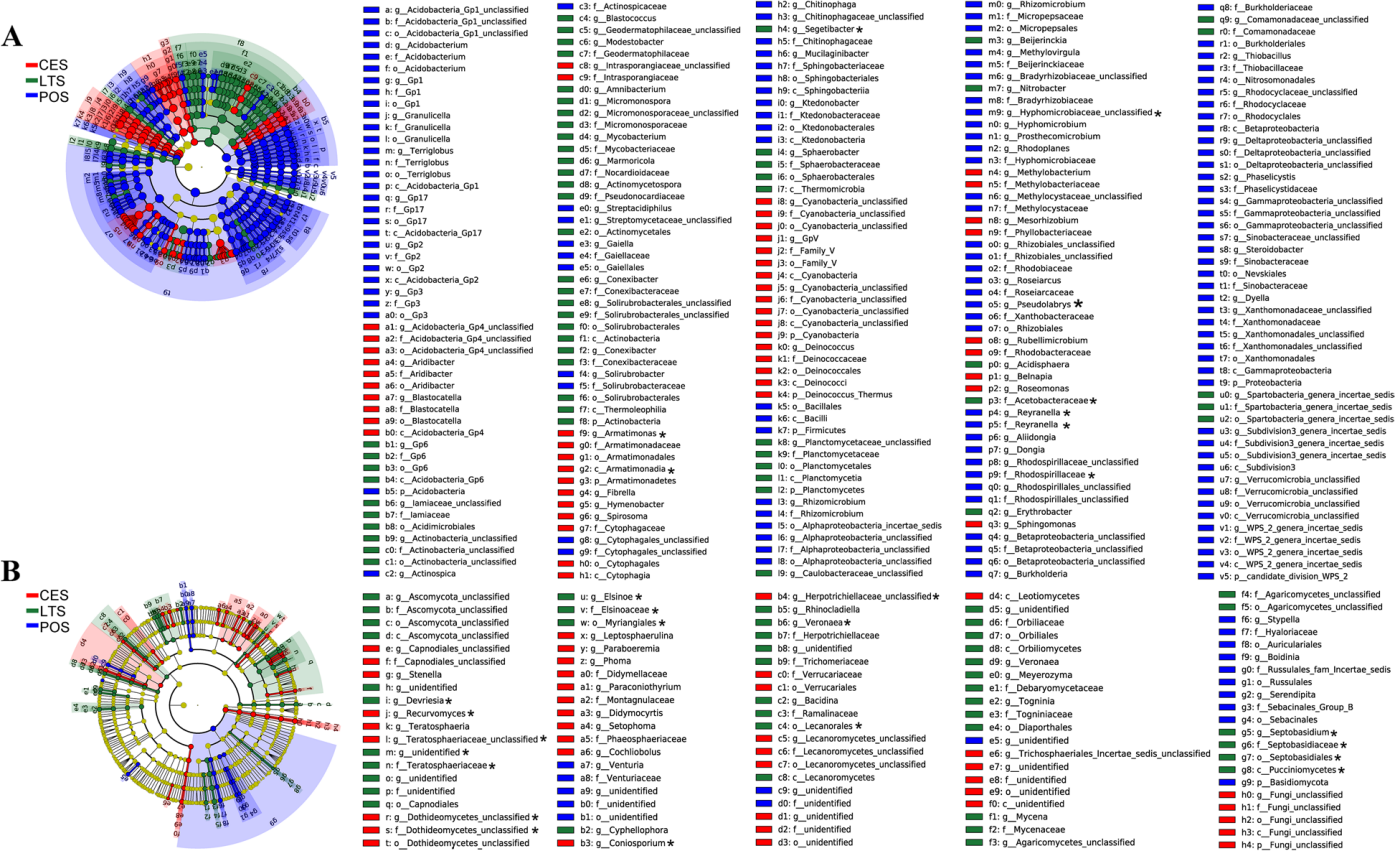
Cladograms showing the phylogenetic distribution of the bacterial (**A**) and fungal (**B**) lineages associated with three kinds of cultivating substrates. Circles indicate phylogenetic levels from domain to genus. The diameter of each circle is proportional to the abundance of the group. Different-colored regions represent different cultivation modes. POS: pine tree bark from PO, CES: rocks from CE, and LTS: pear tree bark from LT. *: the biomarkers shared by the plants and the substrates

A total of 110 bacterial and 135 fungal biomarkers with significant differences were detected in *D. catenatum* plants among the three cultivation modes according to LEfSe analysis (Fig. 4 and Fig. S1). In PO plants, 13 bacterial and 11 fungal groups were significantly enriched, whereas in LT plants, 11 bacterial and 15 fungal groups were significantly enriched, and in CE plants, 12 bacterial and 15 fungal groups were significantly enriched. There were 362 bacterial and 149 fungal biomarkers with significant differences among cultivation substrates (Fig. 5 and Fig. S2). In POS, 48 bacterial and 8 fungal groups were significantly enriched, whereas in LTS, 23 bacterial and 17 fungal groups were significantly enriched, and in CES, 15 bacterial and 17 fungal groups were significantly enriched.

### Correlations between plant chemical components and microbial communities

There were significant differences in stem polysaccharide and ethanol-soluble extractive content, and leaf flavonoid and phenol content in *D. catenatum* plants among the three cultivation modes (Table 1). The highest polysaccharide content was found in stems from CE, followed by those from LT and PO. The ethanol-soluble extractive content was significantly higher in stems from PO than in those from LT and CE. The total flavonoid and phenol contents were higher in leaves from LT and CE than in those from PO.

**Table 1:** The content of main chemical components of *D. catenatum* from three cultivation modes (*x* ± *s*, *n* = 3)

Cultivation mode	Polysaccharides mg/g	Ethanol-soluble extractive mg/g	Flavonoids mg/g	Phenols mg/g
PO	295.40 ± 5.72 Cc	112.23 ± 3.30 Aa	4.37 ± 0.34 Cc	8.69 ± 0.16 Cc
CE	394.77 ± 9.15Aa	85.27 ± 8.05 Bb	8.52 ± 0.29 Bb	13.34 ± 0.17 Aa
LT	336.47 ± 6.96 Bb	95.70 ± 5.11 ABb	10.87 ± 0.30 Aa	12.16 ± 0.35 Bb

The capital means *P* < 0.01; the lowercase means *P* < 0.05

**Fig. 6:**
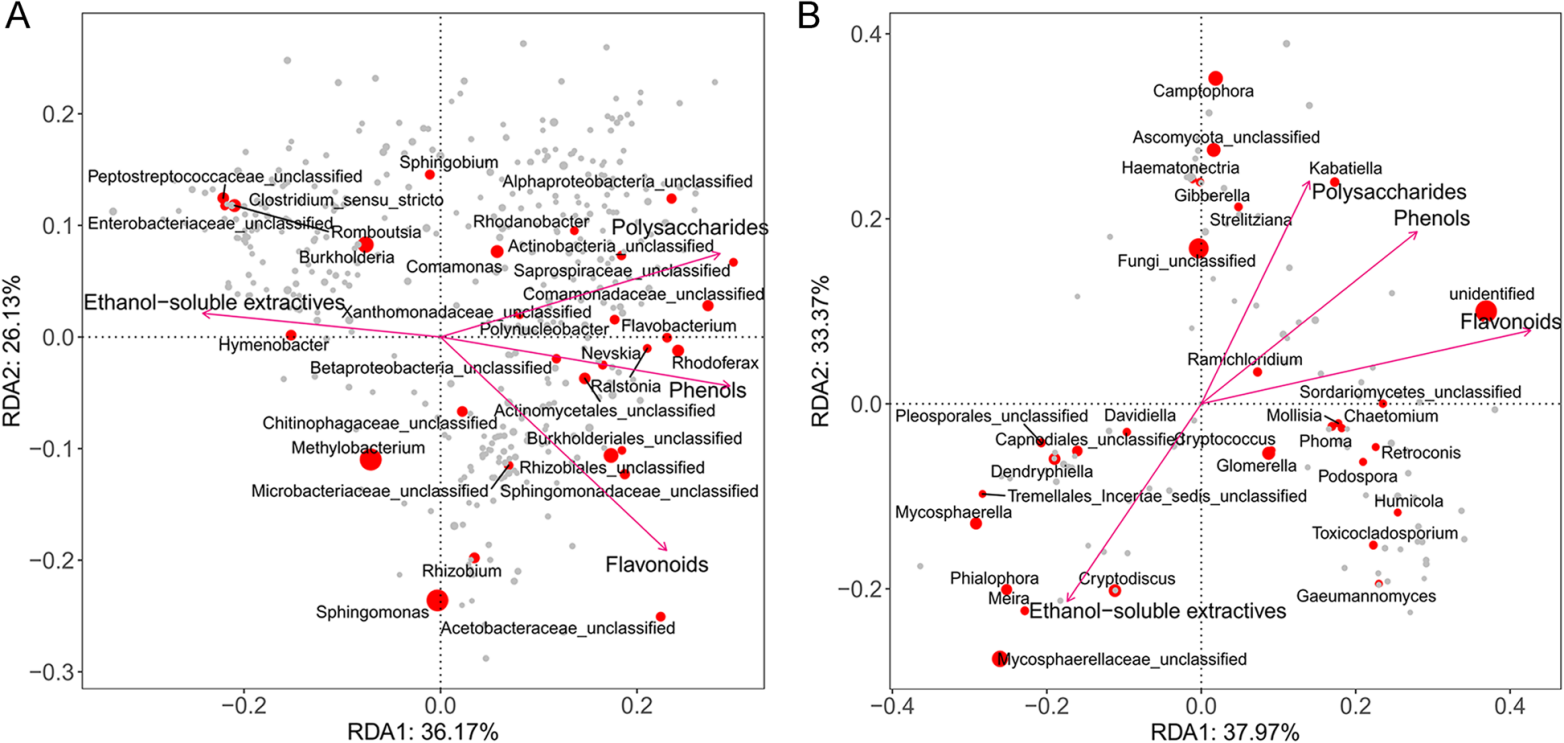
Redundancy analysis (RDA) analysis of bacterial and fungal communities at genus level (symbols) and the content of four main chemical components in *D. catenatum* (arrows). Bacterial and fungal communities are shown in (**A**, **B**), respectively. The values of axes 1 and 2 are the percentages explained by the corresponding axis

The relationships between genus-level microbial communities and plant chemical components were explored using redundancy analysis. The results revealed that several bacterial and fungal genera with high relative abundances (> 1%) were closely related to polysaccharide, ethanol-soluble extractive, flavonoid, and phenol content (Fig. 6). Stem polysaccharide and leaf phenol and flavonoid content were positively correlated with 21 bacterial genera including *Polynucleobacter*, *Rhodanobacter*, *Comamonas*, *Nevskia*, *Ralstonia*, *Rhodoferax*, *Flavobacterium*, *Rhizobium*, and unclassified Xanthomonadaceae, Saprospiraceae, Actinobacteria, Comamonadaceae, Alphaproteobacteria, Betaproteobacteria, Actinomycetales, Sphingomonadaceae, Rhizobiales, Microbacteriaceae, Burkholderiales, Chitinophagaceae, Acetobacteraceae genera, and 15 fungal genera including *Kabatiella*, *Strelitziana*, *Gibberella*, *Haematonectria*, *Camptophora*, *Ramichloridium*, *Chaetomium*, *Mollisia*, *Phoma*, *Retroconis*, and *Podospora* and unclassified Ascomycota and Sordariomycetes genera. Ethanol-soluble extractive content was positively correlated with six bacterial genera, *Hymenobacter*, *Burkholderia*, *Romboutsia*, *Methylobacterium*, and unclassified Enterobacteriaceae and Peptostreptococcaceae genera; and 10 fungal genera, *Cryptodiscus*, *Meira*, *Phialophora*, *Davidiella*, *Dendryphiella*, *Mycosphaerella*, and unclassified Mycosphaerellaceae, Capnodiales, Tremellales, and Pleosporales genera.

## Discussion

In this study, we examined the bacterial and fungal communities of *D. catenatum* ‘Jingpin Tianmushan’ and its substrates from three different cultivation modes using high-throughput sequencing methods. To our knowledge, this study is the first to characterize bacterial and fungal communities from *D. catenatum* and its cultivation substrates; our results indicate a complex relationship between this orchid and its associated microbial communities under different cultivation conditions.

### Bacteria and fungi performed different abundance, diversity and community structure dependent on cultivation mode

The abundance and diversity of bacteria and fungi varied according to the cultivation mode. The abundances and diversity index of bacterial communities were significantly lower in CES than in POS, whereas those of fungal communities were barely affected by cultivation mode. However, in plant organs, the fungal abundance and diversity were significantly higher in CE than in PO, whereas those of bacterial communities were barely affected by cultivation mode. Bacteria appeared to be more sensitive to cultivating substrate than fungi, which is inconsistent with the findings of Trivedi et al. [28], that fungal establishment in the rhizosphere and on plant roots was affected to a greater extent by stochastic variation and responded differently than bacteria to environmental factors. These contrasting responses of bacteria and fungi between studies may be related to differences in the soil types, plant species, and/or plant organs investigated [1]. Rock is normally more barren than bark, and therefore may be associated with less microbial diversity, but such harsh environmental conditions may be conducive to accumulating microbial communities associated with plants. Andrade et al. [29] hypothesized that plant microbiota are facilitators that provide a suitable environment for plant hosts, and are involved in plant adjustment to local biotic or abiotic ecosystem conditions. Bacterial richness and fungal richness and diversity were all significantly higher in roots than in leaves from PO, whereas bacterial richness and diversity were significantly higher in leaves and roots than in stems from CE. These results indicate that the richness and diversity of plant-associated microbiota are influenced by both the cultivation environment and plant organ [28, 30].

Bacteria and fungi showed different community structure responses to different cultivation modes. Boundaries were more distinct among bacterial groups than fungal groups in plants and their substrates (Fig. 2). This result also supports our speculation that bacteria may be more susceptible to cultivation environments. Differential occurrence of microbes associated with plants is likely related to biotic and abiotic constraints determined by their sessile lifestyle [29].

Both bacterial and fungal community structures differed significantly between PO and the other two cultivation modes, but not between LT and CE, in both plants and their substrates. Microbial communities are influenced by environmental characteristics [31], including soil factors such as soil type, moisture, texture, pH, and nutrient availability [32]. The main difference between PO and the other two modes (LT and CE) is that PO is indoors. The bacterial and fungal community structures of the LT and CE substrates were more similar because both are outdoors. Factors that can differ between indoor and outdoor environments include temperature, moisture, and light intensity [25]; these factors may have led to differences in bacterial and fungal community structure between POS and the two outdoor cultivation substrates.

Regardless of the cultivation mode, both bacterial and fungal community structures were influenced by plant organ, with significant differences between roots and the other two organs examined (stems and leaves), but not between stems and leaves. Plant compartment is a major selective force shaping the composition of plant-associated microbiota [28]. Several studies reported that there are clear differences in microbial communities among the rhizosphere, leaf and root endosphere, phyllosphere, and the bulk and root-zone soils [33]. Our study indicated that the bacterial and fungal community structures in roots were similar to those of their substrates. Also, venn diagrams (Fig. S3) showed that subsets of bacterial and fungal OTUs were shared by the three plant organs and their substrates, and that most bacterial and fungal OTUs were shared among the roots and their substrates. These results support the conclusion that although assemblies of root-associated bacteria and fungi differ substantially from aboveground communities, both represent a subset of the microbiota derived from soil communities that is enriched in different plant-associated niches [28, 33–35]. A few bacterial and fungal biomarkers, with significant differences among plants or substrates based on linear effect size analysis, were common between plants and substrates (Fig. 4, Fig. 5 and Figs. S1 and S2). Whether these differences in the bacterial and fungal communities of the substrates caused differences in the plant bacterial and fungal communities among cultivation modes requires further investigation.

### Effect of cultivation mode on the compositions of dominant bacterial and fungal communities from *D. catenatum* and its cultivating substrates

The dominant bacterial and fungal communities varied among *D. catenatum* plants and their cultivation substrates, with significant differences observed among bacterial groups, but not among fungal groups. *D. catenatum* was predominantly associated with Proteobacteria, Actinobacteria, Bacteroidetes, and Firmicutes, which was consistent with the findings of previous studies that these were the four most dominant bacterial phyla in association with *Dendrobium* species [36]. Among these dominant phyla, Firmicutes (dominanted by *Romboutsia* and unclassified Peptostreptococcaceae genus) was much more abundant in the leaves and stems of *D. catenatum* from PO than in those from the other two cultivation modes. Firmicutes is the most important beneficial bacterial taxa in ecosystems worldwide [37]. Positive relationships between *Romboutsia* and unclassified Peptostreptococcaceae genera and ethanol-soluble extractive content were found in present study (Table S1). Thus, its enrichment in the leaves and stems of *D. catenatum* from PO may benefit the plant ethanol-soluble extractive accumulation in this cultivation mode.

The predominant bacterial phyla across all cultivating substrates were Proteobacteria, Actinobacteria, Acidobacteria, and Bacteroidetes. This result was consistent with previous studies of soil and plant microbes [38, 39]. Cyanobacteria was the most predominant phylum only in CES, which is expected because many cyanobacteria are known to tolerate environmental extremes such as rock surfaces [40]. The predominant phyla in POS were Proteobacteria and Acidobacteria, whereas those in LTS were Actinobacteria and Proteobacteria. Although POS and LTS are both tree-based substrates, their cultivation environments and tree bark types may have led to variation in their bacterial communities.

The dominant fungal phyla were Ascomycota and Basidiomycota. Ascomycota was predominant across all the samples. These results were consistent with those of previous fungal community studies [30]. However, differences in relative abundance were observed among fungal communities at lower taxonomic levels (Fig. 3D). Agaricomycetes, predominated by Agaricales, Sebacinales, and Trechisporales, were more abundant in roots and substrates from PO than in those from CE. Sebacinales preferred roots, whereas Agaricales preferred bark substrates. Trechisporales preferred PO roots and substrates and CE substrates. These preferences are probably related to their growth habits. Sebacinales species, such as *Piriformospora indica* and *Sebacina vermifera*, are often associated with plant roots [41]. Agaricales includes gilled mushrooms that occur mainly in soil, decayed leaves, and wood [42]. Trechisporales includes fungi that inhabit roots and soil [43].

### Certain bacteria and fungi related to the content of main chemical components in *D. catenatum*

There were significant differences in the main components of *D. catenatum* plants among different cultivation modes. Stem polysaccharide, leaf total flavonoid, and total phenol content were significantly higher in CE and LT plants than in PO plants, whereas stem ethanol-soluble extractive content was significantly higher in PO plants than in CE and LT plants. These results were consistent with the significant differences detected in bacterial and fungal communities between PO and the other two modes (LT and CE) and indicate strong correlations between the plant chemical components and bacterial and fungal communities. The redundancy analysis results also showed that 21 bacterial and 15 fungal genera were positively correlated with stem polysaccharide and leaf phenol and flavonoid content and six bacterial and 10 fungal genera were positively correlated with ethanol-soluble extractive content (Table S1 and S2). Most of them were dominant genera and their relative abundances were closely related to the cultivation modes. The bacteria and fungi positively correlated with stem polysaccharide, leaf total flavonoid, and total phenol content were more abundant in the stems or leaves from CE and LT than in those from PO, while those positively correlated with stem ethanol-soluble extractive content were more abundant in the stems or leaves of PO than in those from the other two cultivation modes. Some of them include beneficial species. For examples, *Rhodanobacter*, *Comamonas*, *Ramichloridium* and *Burkholderia* have been reported as growth-promoting endophytes, Acetobacteraceae and *Rhizobium* members are well-known growth promoting and nitrogen-fixing endophytes, *Chaetomium*, *Mycosphaerella* and *Phoma* endophytes have been reported to produce bioactive metabolites, and some species of *Methylobacterium*, *Phialophora*, *Meira*, and *Dendryphiella* could enhance plant resistance. These bacterial and fungal groups may have great potentials for future application in *D. catenatum* cultivation.

The accumulation of plant metabolic components is influenced by environmental factors. Abiotic factors such as extreme temperature, humidity, and strong light in CE and LT are beneficial to the accumulation of metabolic components in *D. catenatum* [25]. The plant microbiome is an important determinant of plant health and productivity, and has received substantial attention in recent years as a subject of scientific and commercial interest. The microbiome of medicinal plants may directly impact the host metabolites [3, 27]. Therefore, we speculated that variation in the content of the main chemical components among cultivation modes may be affected by the combined effects of abiotic factors and microbial flora. The microbial communities within the substrates mainly affected the microbial communities in the roots of *D. catenatum* plants. Plants may recruit specific microbes from different cultivation substrates; after entering the plant, these microbes are restricted within plant organs, where their accumulation may be affected by the external environment. Thus, the composition and content of metabolic components may interact among different plant organs; these interactions should be explored in a future study.

## Conclusion

We found that cultivation mode impacts microbial communities of *D. catenatum* plants and their substrates. However, the plants and their cultivating substrates exhibited different patterns of bacterial and fungal composition. Plants and substrates differed in dominant bacterial groups, such as Firmicutes and Acidobacteria, but shared dominant fungal groups (Ascomycota and Basidiomycota). Moreover, we observed that bacteria and fungi have different performances dependent on cultivation mode and plant organ. Furthermore, the differences in bacterial and fungal communities between PO and the other two modes (LT and CE) were consistent with those in the content of four main components (polysaccharides, ethanol-soluble extractives, phenols, and flavonoids). Stem polysaccharide and leaf phenol and flavonoid content were positively correlated with 21 bacterial and 15 fungal genera and ethanol-soluble extractive content was positively correlated with six bacterial and 10 fungal genera. It could be of great importance to further elucidate the functions of these bacterial and fungal groups for future application in *D. catenatum* cultivation.

## Materials and methods

### Sample collection

*D. catenatum* variety ‘Jingpin Tianmushan ’, numbered Zhejiang SSVDC0112018, was cultivated in a field located in the mountain town of Tianmu, Lin’an, Zhejiang Province, China (30°20′30″N, 119°26′11″E, 280 m elevation). The study site is the northern margin of the natural distribution area of *D. catenatum*, with a mid-latitude north subtropical monsoon climate. The annual average temperature is 16.6 °C, with 158 precipitation days and 237 frost-free days. The maximum and minimum temperatures occur in July and January, respectively.

*D. catenatum* ‘Jingpin Tianmushan’ was cultivated under the LT, PO, and CE modes as previously described [25]. Biennial leaves, stems, and roots of three *D. catenatum* plants were collected from each cultivation mode for microbial analyses and the determination of main chemical components. We simultaneously collected five samples of the substrate from each cultivation mode: pine tree bark from PO (POS), rocks from CE (CES), and pear tree bark from LT (LTS). Plant samples were rinsed five times with sterile Millipore water. A total of 42 samples, including 27 plant samples and 15 substrate samples, were added to sterile centrifuge tubes and immediately placed in a liquid nitrogen canister, transferred to the laboratory, and stored in a freezer at − 80 °C for microbial analyses.

### Determination of main chemical components

*D. catenatum* stem and leaf samples from each mode were dried to a constant weight at 60 °C in an oven and pulverized, respectively. Polysaccharides were extracted from the stems with distilled water (2 mg/mL DW) in a water bath at 100 °C for 110 min. The stem ethanol-soluble fraction was extracted with 95% ethanol (20 mg/mL DW) in a water bath at 80 °C for 60 min. Total flavonoids and total phenols were extracted from the leaves with 80% methanol (20 mg/mL DW) and sonicated in an ultrasonic water bath at 100 W and 25 °C for 30 min as previously described [44].

Polysaccharide content was determined following the phenol-sulfuric acid method using glucose as a standard according to the method described in the Chinese Pharmacopoeia [18]. The content of ethanol-soluble extractives was determined using the hot-dipping method, as described in the Chinese Pharmacopoeia [18]. Total flavonoid and phenols content were determined using ultraviolet spectrophotometry with rutin and gallic acid as the reference standards, respectively [45, 46].

### DNA extraction

DNA was extracted from plant samples using the cetyltrimethylammonium bromide (CTAB) method. DNA was extracted from POS and LTS samples using a soil DNA kit (OMEGA Bio-tek, Norcross, GA, USA), according to the manufacturer’s instructions. CES samples were dissolved in sterile Millipore water, followed by DNA extraction using a Water DNA kit (OMEGA Bio-tek), according to the manufacturer’s instructions. These reagents were designed to extract DNA from trace amounts in samples, and have been shown to be effective in preparing DNA from most microbes. Nuclease-free water was used as a blank. Total DNA was eluted in 50 μL of elution buffer and stored at − 80 °C.

### Polymerase chain reaction (PCR) amplification and rDNA sequencing

The V4 region of the 16S rRNA gene was amplified using the primers fM1/rC5 and 515f-GC/rC5 [47]. The internal transcribed spacer 2 (ITS2) region of the eukaryotic (fungi) small-subunit rRNA gene was amplified using slightly modified versions of primers fITS7 and ITS4 [48]. The 5′ ends of the primers were tagged with a specific barcode for each sample. PCR amplification was performed in 25 μL of reaction mixture containing 25 ng of template DNA, 12.5 μL of PCR Premix, 2.5 μL of each primer, and PCR-grade water to adjust the volume. The PCR conditions for amplification of the eukaryotic ITS and prokaryotic 16S fragments consisted of initial denaturation at 98 °C for 30 s; 35 cycles of denaturation at 98 °C for 10 s, annealing at 54 °C/52 °C for 30 s, and extension at 72 °C for 45 s; followed by a final extension at 72 °C for 10 min. The PCR products were confirmed via 2% agarose gel electrophoresis.

The PCR products were purified using AMPure XT beads (Beckman Coulter Genomics, Danvers, MA, USA) and quantified using a fluorometer (Qubit, Invitrogen, Carlsbad, CA, USA). The amplicon pools were prepared for sequencing and the size and quantity of the amplicon library were assessed using an Agilent 2100 Bioanalyzer (Agilent, Santa Clara, CA, USA) and the Library Quantification Kit for Illumina (Kapa Biosciences, Woburn, MA, USA), respectively. The PhiX Control library (v3) (Illumina, San Diego, CA, USA) was combined with the amplicon library (expected at 30%).

### Sequence analysis

The libraries were sequenced using the Illumina 250PE MiSeq platform, according to the manufacturer’s recommendations. Paired-end reads were assigned to samples based on their unique barcodes and truncated by cutting off the barcode and primer sequence. Paired-end reads were merged using the FLASH (16S, Version 1.2.8) and PEAR (ITS, Version 0.9.6) programs. Quality filtering of the raw tags was performed under specific filtering conditions to obtain clean, high-quality tags using the FastQC program (Version 0.10.1). Chimeric sequences were filtered using the Vsearch software (Version 2.3.4). Sequences with 97% similarity were assigned to the same operational taxonomic units (OTUs) using the Vsearch software (Version 2.3.4). All OTUs consisting of one single sequence (singletons) and sequence with lower abundance value (< 0.001% of total sequence) were removed [49]. Representative sequences were chosen for each OTU, and taxonomic data were then assigned to each representative sequence. Bacterial taxonomic data were assigned using the RDP (http://rdp.cme.msu.edu) and NT-16S databases, and fungal taxonomic data were assigned using the RDP and UNITE (https://unite.ut.ee) databases. Differences among the dominant species in each group after multiple sequence alignment were evaluated using the PyNAST software, which examines the phylogenetic relationships among different OTUs. OTU abundance data were normalized using a standard sequence number corresponding to the sample with the fewest sequences. The sequence data are available on the National Center for Biotechnology Information BioSample database (accession numbers SAMN18533069 - SAMN18533110, SAMN18530906 - SAMN18530947).

### Statistical analysis

Alpha diversity was calculated with QIIME software (Version 1.8.0) based on Chao1, Shannon, Simpson and Observed species indices. Beta diversity analysis was used to evaluate differences in species complexity within samples. Beta diversity was calculated by QIIME software (Version 1.8.0) and evaluated by principle co-ordinates analysis (PCoA). Cluster analysis and stacked bar charts were used to compare bacterial and fungal community compositions in the plants and substrates. According to the resulting species abundance and species annotations, the relative abundances of the 20 taxa with the highest abundance at the phylum and class levels for each sample were calculated and stacked bar charts were created. Cluster analysis of plant and substrate samples was performed based on the Bray–Curtis dissimilarity matrix.

Differences in alpha diversity indices among cultivation modes or plant organs were analyzed using non-parametric Mann-Whitney U test, and main chemical components of the samples were analyzed using one-way analysis of variance (ANOVA) in the IBM SPSS Statistics software (v24.0; IBM Corp., Armonk, NY, USA). Significant differences were evaluated using the least significant difference (LSD) test at a level of *P* < 0.05. Linear discriminant analysis (LDA) was coupled with effect size analysis (LEfSe) to search for statistically different biomarkers between groups [50]. In brief, the non-parametric factorial Kruskal-Wallis (KW) sum-rank test was used to detect microbes with significant differential abundance (*P <* 0.05) with respect to the class. Then, biological consistency was subsequently investigated using a set of pairwise tests among subclasses using the (unpaired) Wilcoxon rank-sum test. As last, LEfSe used LDA score to estimate the effect size of each differentially abundant microbe. The microbes with LDA score ≥ 3 were confirmed as biomarkers by linear effect size analysis. LEfSe was performed using the OmicStudio online tool (https://www.omicstudio.cn/tool/). The relationships among plant bacterial and fungal community structures and chemical components were analyzed using redundancy analysis and variation partitioning (https://bioincloud.tech/#/task-ui/rda), which eliminates redundant variables depending on other measured variables, automatically selecting variables with large effects, to gradually remove redundant parameters; significance levels are based on 999 Monte Carlo permutations [51, 52].

## Supplementary Information

**Additional file 1: Fig. S1.** Indicator bacteria with LDA scores of 3 or greater in bacterial (A) and fungal (B) communities associated with *D. catenatum* from three different modes. CE: cliff epiphytic cultivation, LT: living tree epiphytic cultivation, PO: Pot cultivation. Different-colored regions represent different cultivation modes. *: the biomarkers shared by the plants and the substrates.

**Additional file 2: Fig. S2.** Indicator bacteria with LDA scores of 3 or greater in bacterial (A) and fungal (B) communities from three different cultivating substrates. POS: pine tree bark from PO, CES: rocks from CE, and LTS: pear tree bark from LT. Different-colored regions represent different cultivation modes. *: the biomarkers shared by the plants and the substrates.

**Additional file 3: Fig. S3.** Venn diagram to indicate number of shared and unique bacterial and fungal OTUs identified in three plant compartments of *D. catenatum* and three substrates from different cultivation modes. Each ellipse represents a compartment or a kind of substrates from a cultivation mode. CE: cliff epiphytic cultivation, LT: living tree epiphytic cultivation, PO: Pot cultivation, POS: pine tree bark from PO, CES: rocks from CE, and LTS: pear tree bark from LT.

**Additional file 4: Table S1.** Relative abundances of the bacterial genera that correlated with the content of main chemical components in leaves and stems of *D. catenatum* from three cultivation modes. **Table S2.** Relative abundances of the fungal genera that correlated with the content of main chemical components in leaves and stems of *D. catenatum* from three cultivation modes.

## Data Availability

The sequence data are available on the National Center for Biotechnology Information BioSample database (accession numbers SAMN18533069 - SAMN18533110, SAMN18530906-SAMN18530947).

## Abbreviations

*LT*: living tree epiphytic cultivation
*CE*: cliff epiphytic cultivation
*PO*: pot cultivation
*C13*: *D. catenatum* ‘Jingpin Tianmushan’
*POS*: pine tree bark from PO
*CES*: rocks from CE
*LTS*: pear tree bark from LT
*CTAB*: Cetyltrimethylammonium bromide
*PCR*: Polymerase chain reaction
*ITS*: Internal Transcribed Spacer
*OTU*: Operational Taxonomic Unit
*PCoA*: Principle co-ordinates analysis
*ANOVA*: One-way analysis of variance
*LSD*: Least significant difference
*LDA*: Linear discriminant analysis
*LEfSe*: Linear discriminant analysis Effect Size
*KW*: Kruskal-Wallis
