# Supplementary figures and images for: Effect of cultivation mode on bacterial and fungal communities of Dendrobium catenatum
Source: BMC Microbiol. 2022 Sep 21;22:221. doi: 10.1186/s12866-022-02635-6 (PMC9490927; doi:10.1186/s12866-022-02635-6)

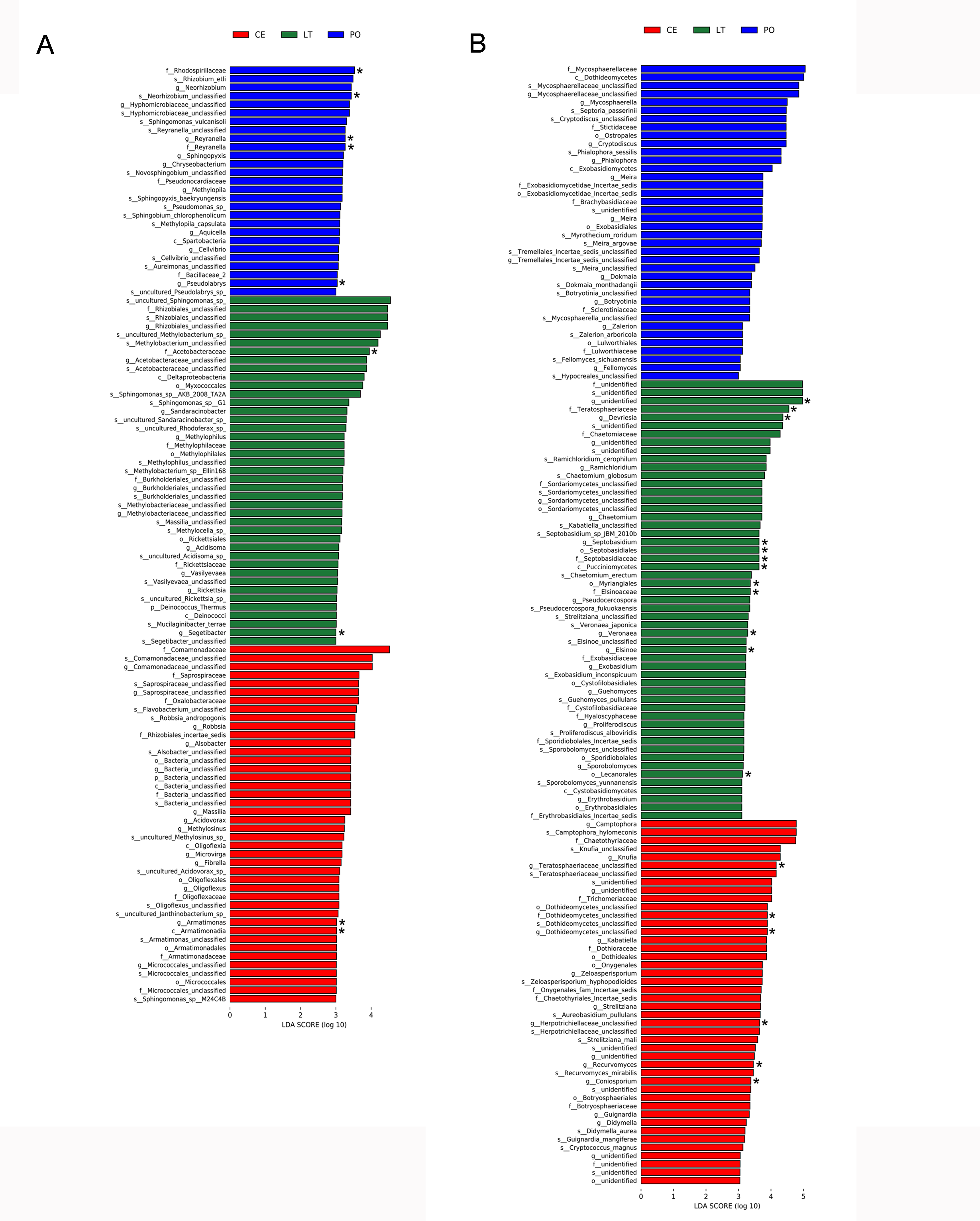

Supplement: Supplementary file 1 — Additional file 1: Fig. S1. Indicator bacteria with LDA scores of 3 or greater in bacterial (A) and fungal (B) communities associated with D. catenatum from three different modes. CE: cliff epiphytic cultivation, LT: living tree epiphytic cultivation, PO: Pot cultivation. Different-colored regions represent different cultivation modes. *: the biomarkers shared by the plants and the substrates. [file 12866_2022_2635_MOESM1_ESM.tif]

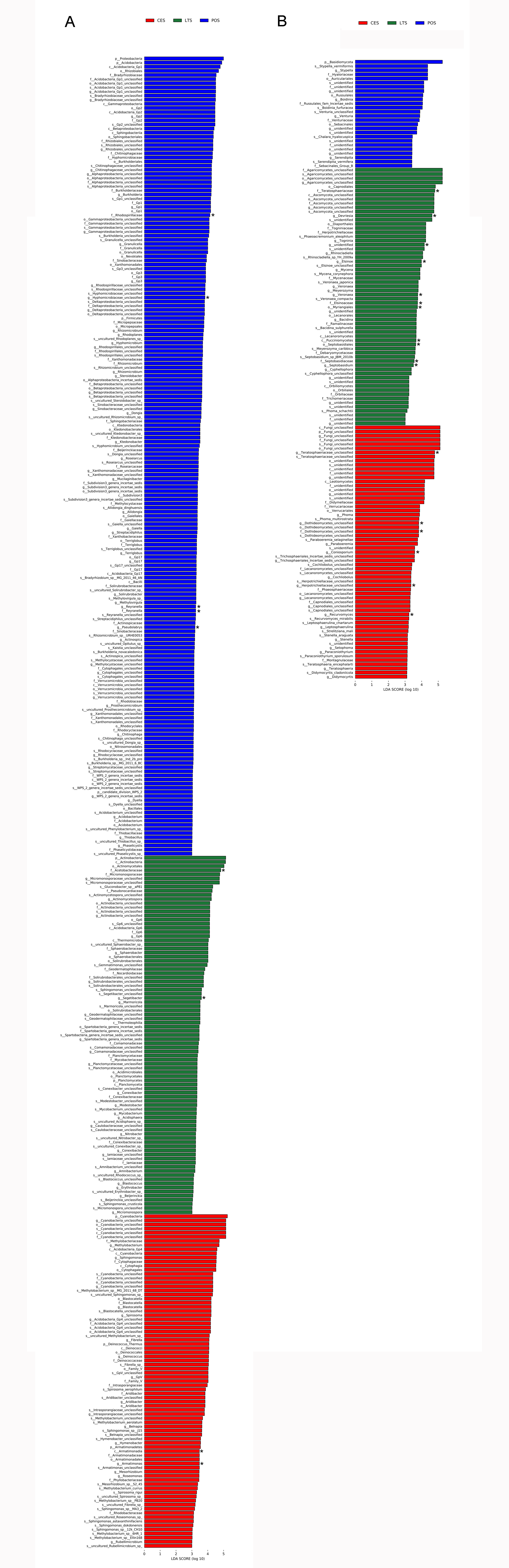

Supplement: Supplementary file 2 — Additional file 2: Fig. S2. Indicator bacteria with LDA scores of 3 or greater in bacterial (A) and fungal (B) communities from three different cultivating substrates. POS: pine tree bark from PO, CES: rocks from CE, and LTS: pear tree bark from LT. Different-colored regions represent different cultivation modes. *: the biomarkers shared by the plants and the substrates. [file 12866_2022_2635_MOESM2_ESM.tif]

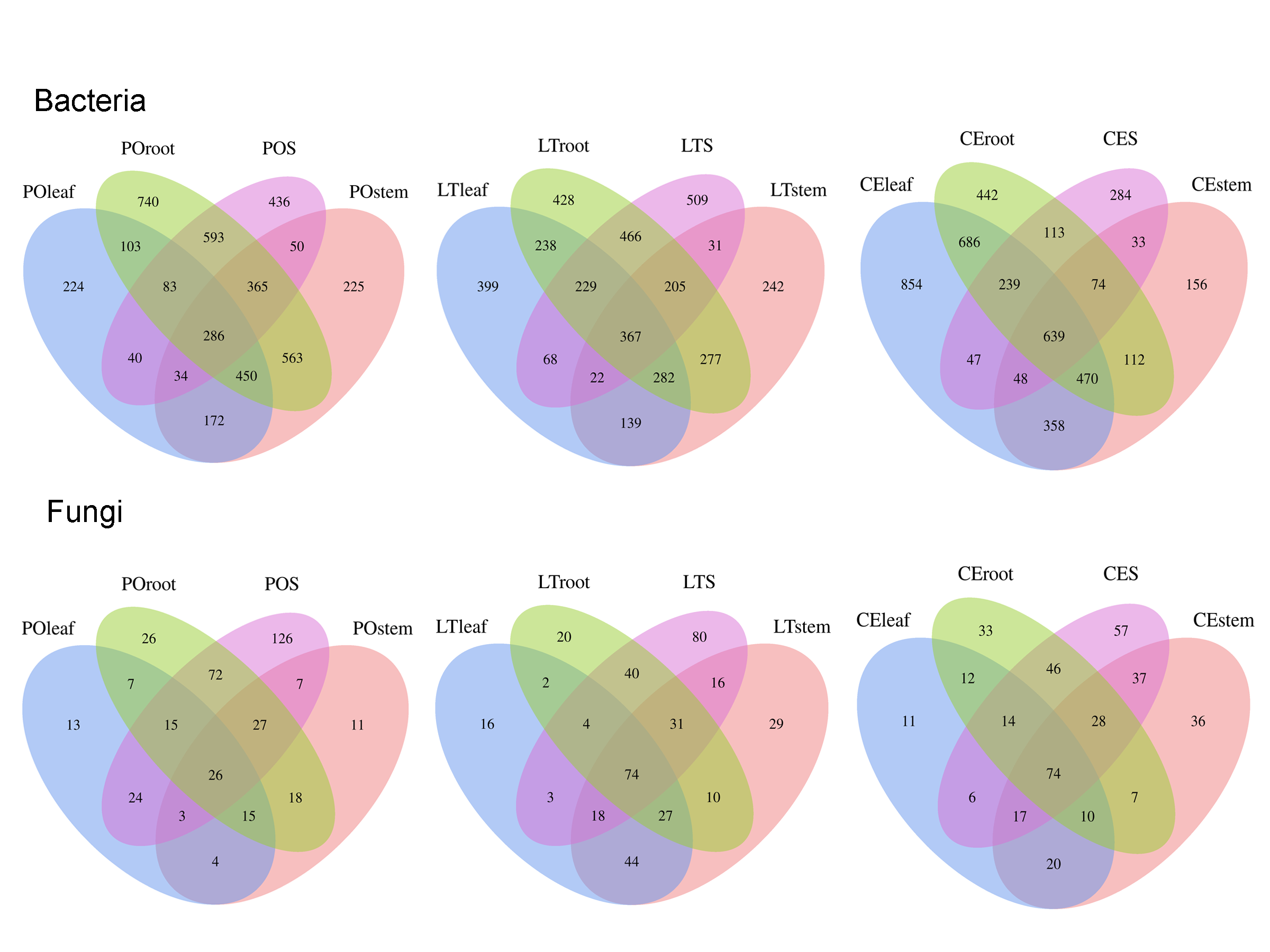

Supplement: Supplementary file 3 — Additional file 3: Fig. S3. Venn diagram to indicate number of shared and unique bacterial and fungal OTUs identified in three plant compartments of D. catenatum and three substrates from different cultivation modes. Each ellipse represents a compartment or a kind of substrates from a cultivation mode. CE: cliff epiphytic cultivation, LT: living tree epiphytic cultivation, PO: Pot cultivation, POS: pine tree bark from PO, CES: rocks from CE, and LTS: pear tree bark from LT. [file 12866_2022_2635_MOESM3_ESM.tif]
