# Supplementary material for: Effect of cultivation mode on bacterial and fungal communities of Dendrobium catenatum
Source: BMC Microbiol. 2022 Sep 21;22:221. doi: 10.1186/s12866-022-02635-6 (PMC9490927; doi:10.1186/s12866-022-02635-6)
Supplement: Supplementary file 4 — Additional file 4: Table S1. Relative abundances of the bacterial genera that correlated with the content of main chemical components in leaves and stems of D. catenatum from three cultivation modes. Table S2. Relative abundances of the fungal genera that correlated with the content of main chemical components in leaves and stems of D. catenatum from three cultivation modes. [file 12866_2022_2635_MOESM4_ESM.docx]

**Supporting information**

Table S1 Relative abundances of the bacterial genera that correlated with the content of key chemical components in leaves and stems of *D. catenatum* from three cultivation modes

| Taxonomy | POleaf | POstem | CEleaf | CEstem | LTleaf | LTstem |
| --- | --- | --- | --- | --- | --- | --- |
| Acetobacteraceae_unclassified | 0.23% | 0.07% | 0.91% | 1.23% | 1.77% | 1.87% |
| Actinobacteria_unclassified | 0.39% | 0.51% | 0.87% | 2.07% | 1.19% | 0.21% |
| Actinomycetales_unclassified | 1.25% | 1.09% | 3.90% | 1.22% | 4.52% | 0.47% |
| Alphaproteobacteria_unclassified | 0.72% | 0.57% | 2.64% | 1.24% | 1.72% | 0.58% |
| Betaproteobacteria_unclassified | 0.91% | 0.14% | 1.13% | 1.09% | 0.88% | 0.81% |
| Burkholderiales_unclassified | 0.57% | 0.03% | 0.62% | 0.55% | 0.95% | 0.61% |
| Chitinophagaceae_unclassified | 1.21% | 1.49% | 2.22% | 0.60% | 2.49% | 1.06% |
| Comamonadaceae_unclassified | 0.62% | 0.28% | 4.46% | 1.50% | 3.38% | 0.64% |
| *Comamonas* | 5.11% | 0.10% | 2.34% | 6.48% | 3.63% | 0.15% |
| *Flavobacterium* | 0.24% | 0.05% | 2.30% | 1.07% | 1.88% | 0.61% |
| Microbacteriaceae_unclassified | 0.93% | 0.48% | 1.28% | 0.43% | 0.81% | 1.49% |
| *Nevskia* | 0.60% | 0.01% | 1.67% | 0.57% | 2.01% | 0.00% |
| *Polynucleobacter* | 0.24% | 0.01% | 2.24% | 0.97% | 2.27% | 0.29% |
| *Ralstonia* | 0.05% | 0.01% | 1.09% | 0.96% | 1.42% | 0.00% |
| Rhizobiales_unclassified | 1.28% | 1.37% | 4.16% | 6.27% | 4.14% | 11.39% |
| *Rhizobium* | 0.33% | 0.54% | 0.59% | 0.50% | 0.10% | 7.33% |
| *Rhodanobacter* | 0.00% | 0.00% | 0.02% | 3.43% | 0.00% | 0.00% |
| *Rhodoferax* | 0.25% | 0.06% | 5.67% | 2.10% | 5.09% | 0.66% |
| Saprospiraceae_unclassified | 0.00% | 0.02% | 1.36% | 1.06% | 0.86% | 0.16% |
| Sphingomonadaceae_unclassified | 0.52% | 0.90% | 0.92% | 2.21% | 1.53% | 1.55% |
| Xanthomonadaceae_unclassified | 0.69% | 0.32% | 2.88% | 0.22% | 0.53% | 0.33% |
| *Burkholderia* | 0.21% | 23.26% | 2.50% | 10.11% | 1.50% | 0.46% |
| Enterobacteriaceae_unclassified | 2.02% | 1.62% | 0.03% | 0.36% | 0.40% | 0.03% |
| *Hymenobacter* | 4.19% | 1.15% | 0.49% | 1.25% | 1.81% | 0.36% |
| *Methylobacterium* | 21.58% | 11.44% | 6.63% | 15.47% | 24.37% | 10.47% |
| Peptostreptococcaceae_unclassified | 5.81% | 6.73% | 0.00% | 0.00% | 0.01% | 0.00% |
| *Romboutsia* | 7.30% | 10.33% | 0.02% | 0.01% | 0.18% | 0.23% |

Note: the relative abundances of the bacterial genera with yellow background are positively correlated with stem polysaccharide and leaf phenol and flavonoid content; the relative abundances of the bacterial genera with blue background are positively correlated with ethanol-soluble extractive content.

Table S2 Relative abundances of the fungal genera that correlated with the content of key chemical components in leaves and stems of *D. catenatum* from three cultivation modes

| Taxonomy | POleaf | POstem | CEleaf | CEstem | LTleaf | LTstem |
| --- | --- | --- | --- | --- | --- | --- |
| Ascomycota_unclassified | 0.07% | 5.71% | 10.76% | 7.44% | 3.61% | 4.35% |
| *Camptophora* | 0.00% | 0.01% | 24.64% | 12.13% | 1.64% | 0.74% |
| *Chaetomium* | 0.00% | 0.00% | 0.00% | 0.20% | 3.17% | 0.23% |
| *Gibberella* | 0.01% | 0.08% | 0.04% | 3.72% | 0.26% | 0.08% |
| *Haematonectria* | 0.00% | 0.00% | 0.01% | 2.78% | 0.00% | 0.03% |
| *Kabatiella* | 0.00% | 0.00% | 0.66% | 3.59% | 1.69% | 1.91% |
| *Mollisia* | 0.00% | 0.00% | 0.00% | 0.00% | 0.02% | 3.36% |
| *Phoma* | 0.00% | 0.03% | 0.10% | 0.17% | 0.05% | 5.25% |
| *Podospora* | 0.00% | 0.00% | 0.00% | 0.04% | 2.62% | 0.53% |
| *Ramichloridium* | 0.90% | 1.00% | 0.12% | 0.19% | 1.43% | 2.95% |
| *Retroconis* | 0.00% | 0.00% | 0.01% | 0.31% | 2.00% | 0.60% |
| Sordariomycetes_unclassified | 0.00% | 0.00% | 0.00% | 1.05% | 2.00% | 1.03% |
| *Strelitziana* | 0.05% | 0.00% | 1.57% | 2.12% | 0.16% | 0.94% |
| Capnodiales_unclassified | 6.82% | 0.57% | 1.04% | 1.32% | 0.61% | 2.13% |
| *Cryptodiscus* | 0.00% | 24.00% | 0.01% | 0.00% | 0.00% | 0.00% |
| *Davidiella* | 1.07% | 0.80% | 0.24% | 1.09% | 0.50% | 0.60% |
| *Dendryphiella* | 0.00% | 19.56% | 0.00% | 0.00% | 0.03% | 0.01% |
| *Meira* | 4.62% | 0.69% | 0.02% | 0.00% | 0.00% | 0.01% |
| *Mycosphaerella* | 9.73% | 9.98% | 0.06% | 0.01% | 0.34% | 0.57% |
| Mycosphaerellaceae_unclassified | 39.60% | 6.69% | 0.03% | 0.06% | 7.06% | 0.08% |
| *Phialophora* | 10.29% | 2.13% | 0.27% | 0.13% | 1.40% | 0.57% |
| Pleosporales_unclassified | 0.00% | 5.56% | 0.00% | 0.45% | 0.03% | 0.26% |
| Tremellales_Incertae_sedis_unclassified | 0.89% | 1.64% | 0.01% | 0.00% | 0.01% | 0.00% |

Note: the relative abundances of the fungal genera with yellow background are positively correlated with stem polysaccharide and leaf phenol and flavonoid content; the relative abundances of the fungal genera with blue background are positively correlated with ethanol-soluble extractive content.
